# Supplementary material for: Transcriptome Analysis Reveals Vimentin-Induced Disruption of Cell–Cell Associations Augments Breast Cancer Cell Migration
Source: Cells. 2022 Dec 13;11(24):4035. doi: 10.3390/cells11244035 (PMC9776984; doi:10.3390/cells11244035)
Supplement: Supplementary file 1 [file cells-11-04035-s001.zip › DEGs and enrichment analysis.zip!/DEGs and enrichment analysis/WT_VIMvsVector KEGG/GSEA/WT_VIM/PHOTOTRANSDUCTION/PHOTOTRANSDUCTION(HSA04744).html]

Details for gene set PHOTOTRANSDUCTION(HSA04744)[GSEA]

|  || Dataset | fpkm.sample |
| Phenotype | sample.cls |
| Upregulated in class | WT\_VIM |
| GeneSet | PHOTOTRANSDUCTION(HSA04744) |
| Enrichment Score (ES) | 0.66058004 |
| Normalized Enrichment Score (NES) | 1.1496696 |
| Nominal p-value | 0.0 |
| FDR q-value | 0.28544328 |
| FWER p-Value | 0.498 |
Table: GSEA Results Summary

  

Fig 1: Enrichment plot: PHOTOTRANSDUCTION(HSA04744)      
 Profile of the Running ES Score & Positions of GeneSet Members on the Rank Ordered List

  

| PROBE | DESCRIPTION (from dataset) | GENE SYMBOL | GENE\_TITLE | RANK IN GENE LIST | RANK METRIC SCORE | RUNNING ES | CORE ENRICHMENT || 1 | ENSG00000242686 | AC107464.1 |  |  | 1963 | Infinity | -0.0329 | Yes |
| 2 | ENSG00000114349 | GNAT1 |  |  | 2937 | 5.217 | 0.1012 | Yes |
| 3 | ENSG00000185527 | PDE6G |  |  | 3364 | 3.849 | 0.2051 | Yes |
| 4 | ENSG00000112599 | GUCA1B |  |  | 4761 | 2.514 | 0.2541 | Yes |
| 5 | ENSG00000132915 | PDE6A |  |  | 4830 | 2.475 | 0.3244 | Yes |
| 6 | ENSG00000160014 | CALM3 |  |  | 5678 | 2.145 | 0.3720 | Yes |
| 7 | ENSG00000178372 | CALML5 |  |  | 6997 | 1.816 | 0.4021 | Yes |
| 8 | ENSG00000127928 | GNGT1 |  |  | 7179 | 1.778 | 0.4504 | Yes |
| 9 | ENSG00000108370 | RGS9 |  |  | 7973 | 1.627 | 0.4839 | Yes |
| 10 | ENSG00000078369 | GNB1 |  |  | 8886 | 1.494 | 0.5116 | Yes |
| 11 | ENSG00000198668 | CALM1 |  |  | 10296 | 1.325 | 0.5260 | Yes |
| 12 | ENSG00000229097 | CALM2P2 |  |  | 11566 | 1.221 | 0.5398 | Yes |
| 13 | ENSG00000133256 | PDE6B |  |  | 11661 | 1.213 | 0.5733 | Yes |
| 14 | ENSG00000129007 | CALML4 |  |  | 12062 | 1.177 | 0.6005 | Yes |
| 15 | ENSG00000074621 | SLC24A1 |  |  | 12463 | 1.144 | 0.6267 | Yes |
| 16 | ENSG00000260007 | AC107871.1 |  |  | 12480 | 1.143 | 0.6594 | Yes |
| 17 | ENSG00000070729 | CNGB1 |  |  | 14407 | 0.996 | 0.6556 | Yes |
| 18 | ENSG00000178363 | CALML3 |  |  | 15698 | 0.929 | 0.6606 | Yes |
| 19 | ENSG00000143933 | CALM2 |  |  | 17677 | 0.818 | 0.6507 | No |
| 20 | ENSG00000169885 | CALML6 |  |  | 19299 | 0.726 | 0.6442 | No |
| 21 | ENSG00000198515 | CNGA1 |  |  | 24865 | 0.366 | 0.5606 | No |
| 22 | ENSG00000114124 | GRK7 |  |  | 26027 | 0.245 | 0.5481 | No |
| 23 | ENSG00000215482 | CALM2P3 |  |  | 26084 | 0.240 | 0.5541 | No |
| 24 | ENSG00000163914 | RHO |  |  | 28954 | 0.000 | 0.5055 | No |
| 25 | ENSG00000185974 | GRK1 |  |  | 30650 | 0.000 | 0.4768 | No |
| 26 | ENSG00000134183 | GNAT2 |  |  | 31083 | NaN | 0.4698 | No |
| 27 | ENSG00000223467 | CALM1P1 |  |  | 33156 | NaN | 0.4350 | No |
| 28 | ENSG00000130561 | SAG |  |  | 34341 | NaN | 0.4153 | No |
| 29 | ENSG00000257289 | AC025423.5 |  |  | 39504 | NaN | 0.3282 | No |
| 30 | ENSG00000048545 | GUCA1A |  |  | 40590 | NaN | 0.3102 | No |
| 31 | ENSG00000138472 | GUCA1C |  |  | 41822 | NaN | 0.2896 | No |
| 32 | ENSG00000278388 | AC002076.2 |  |  | 42599 | NaN | 0.2768 | No |
| 33 | ENSG00000233662 | CALM2P4 |  |  | 42841 | NaN | 0.2730 | No |
| 34 | ENSG00000230564 | CALM1P2 |  |  | 45901 | NaN | 0.2215 | No |
| 35 | ENSG00000267170 | CALM2P1 |  |  | 47225 | NaN | 0.1994 | No |
| 36 | ENSG00000256462 | AL732437.1 |  |  | 47957 | NaN | 0.1873 | No |
| 37 | ENSG00000101890 | GUCY2F |  |  | 49190 | NaN | 0.1668 | No |
| 38 | ENSG00000109047 | RCVRN |  |  | 53046 | NaN | 0.1018 | No |
| 39 | ENSG00000132518 | GUCY2D |  |  | 53057 | NaN | 0.1019 | No |
| 40 | ENSG00000204529 | GUCY2EP |  |  | 53321 | NaN | 0.0978 | No |
| 41 | ENSG00000279174 | AC104581.3 |  |  | 56800 | NaN | 0.0392 | No |
| 42 | ENSG00000276109 | AP005019.3 |  |  | 57164 | NaN | 0.0334 | No |
Table: GSEA details [plain text format]

  

Fig 2: PHOTOTRANSDUCTION(HSA04744)      
 Blue-Pink O' Gram in the Space of the Analyzed GeneSet

  

Fig 3: PHOTOTRANSDUCTION(HSA04744): Random ES distribution      
 Gene set null distribution of ES for **PHOTOTRANSDUCTION(HSA04744)**
